# Identification of lncRNAs Associated With Neuroblastoma in Cross-Sectional Databases: Potential Biomarkers

**DOI:** 10.3389/fnmol.2019.00293

**Published:** 2019-12-12

**Authors:** Bharat Prajapati, Mena Fatma, Mahar Fatima, Md Tipu Khan, Subrata Sinha, Prahlad K. Seth

**Affiliations:** ^1^National Brain Research Centre, Gurgaon, India; ^2^Department of Biochemistry, All India Institute of Medical Sciences, New Delhi, India; ^3^Biotech Park, Lucknow, India

**Keywords:** neuroblastoma, relapsed tumor, long non-coding RNA, disseminated tumor cells, mononuclear cells

## Abstract

Long non-coding RNAs (lncRNAs) have emerged as an important regulatory control in biological systems. Though the field of lncRNA has been progressing rapidly, a complete understanding of the role of lncRNAs in neuroblastoma pathogenesis is still lacking. To identify the abrogated lncRNAs in primary neuroblastoma and in the metastasized as well as the relapsed form of neuroblastoma, we analyzed an RNA-seq dataset on neuroblastoma that is available online to identify the lncRNAs that could potentially be contributing to the biology of neuroblastoma. The identified lncRNAs were further scrutinized using a publicly available epigenetic dataset of neuroblastoma and a cancer database. After this cross-sectional study, we were able to identify three significant lncRNAs, *CASC15*, *PPP1R26-AS1*, and *USP3-AS1*, which could serve as potential biomarkers in clinical studies of neuroblastoma pathogenesis.

## Introduction

Neuroblastoma is an extra-cranial tumor of the nervous system. It develops during embryonic or early post-natal life from the sympathetic cells derived from the neural crest (Tsubota and Kadomatsu, 2018). It is the most common tumor found in infants and has a poor prognosis. The average occurrence of tumor is 1 per 10000 births in the United States (Gurney et al., 1995). Neuroblastoma is a highly heterogeneous disease with several subtypes. Depending on the prognosis and metastasis, the tumor can either be a low-risk regressive tumor with better prognosis or a highly chemotherapy-resistant malignant tumor with very poor prognosis (Diede, 2014). Several factors contribute to these subtypes, including patient age at diagnosis, genetic profile, and the molecular characteristics of the tumor.

The majority of neuroblastoma cases show an early stage of onset, with high malignancy of the disease at the time of diagnosis. Neuroblastoma are usually sporadic and rarely familial. About 1–2% of cases are familial and are inherited in an autosomal dominant manner. *PHOX2B* and *ALK* (anplastic lymphoma kinase) genes are attributed to the familial cases (Kiyonari and Kadomatsu, 2015). GWAS (genome-wide associated studies) have shown other genetic variants that are associated with tumor phenotypes, but malignant neuroblastoma has consistently been shown to have high amplification of the *MYCN* oncogene derived from the short arm of chromosome 2 (2p24) (Ribatti et al., 2002).

High *MYCN* amplification is seen in 40% of patients with the advanced stage of the disease as well as in 5–10% of patients with low-stage disease. The *MYCN* copy number signifies the prognosis of the disease. A high copy number above 10 is associated with the advanced stage of the disease and poor prognosis (Buechner and Einvik, 2012). Apart from the *MYCN* copy number, change in the number of chromosomes (aneuploidy) also results in clinical manifestation of the disease. Approximately 55% of neuroblastoma cases have a triploid number of chromosomes (Davidoff, 2010), while the rest have diploid or tetraploid chromosomes. Patients with triploid or near-triploid chromosomes have a better outcome and survival rate (Spitz et al., 2006). Deletion in the genetic material has also been found in the tumor cells of neuroblastoma, which shows the loss of tumor suppressor genes at the locations of deletion sites. Further, deletions of the short arm of chromosome 1 (1p) and the long arm of chromosome 11 (11q) in many cell lines of neuroblastoma have been reported (Davidoff, 2010). Several studies have attributed the loss of tumor suppressor gene *CHD5* to the loss of chromosome 1p in neuroblastoma (Fujita et al., 2008; Davidoff, 2010).

Despite numerous genetic and molecular studies, the mechanisms underlying the development of aggressive and regressive neuroblastoma are not well understood. The application of advanced high-throughput sequencing technologies has shown the possible role of various non-coding RNAs such as miRNA and long non-coding RNA (lncRNAs) in the development of various diseases and disorders including cancer (Chen et al., 2017). Several non-coding RNAs have been reported to play a role in tumor development by inhibiting or altering the expression of tumor suppressor genes and oncogenes. Long non-coding RNAs are RNA transcripts with a molecular size that is usually 200 nucleotides or more that does not code for a protein (Morlando and Fatica, 2018). LncRNAs are transcribed from the intronic as well as the intergenic region in the genome and occasionally from the antisense region of genes. LncRNAs function by modulating the transcription of several genes, both *cis* and *trans* domain. They modulate the processing of mRNAs and control the post-transcriptional processing of several genes. LncRNAs also function as a scaffold by recruiting chromatin-modifying enzymes to regulate local and distant gene expression. Recent research has highlighted the regulatory as well as the pathophysiological role of lncRNAs such as an lncRNA activator of the enhancer domain (LED), which has been shown to activate the enhancer-mediated transcription of P53, a well-known tumor suppressor gene (Fesler et al., 2016). Down-regulations of LED have been shown in breast, androgen insensitive prostate cancer, and colorectal cancer (Fesler et al., 2016; Sanchez Calle et al., 2018), and similarly, another lncRNA, linc p-21, has shown to be down-regulated during the progression of colorectal cancer (Liu et al., 2017).

Though a few studies have highlighted mutation in genes in neuroblastoma, the role of lncRNAs has not been completely defined. Also, the primary tumor that develops in neuroblastoma can become highly malignant; these tumor cells can migrate to other regions of the body and form disseminated tumors (DTCs). More than 90% of patients with a malignant tumor have disseminated tumor cells that have migrated to the bone marrow at the time of diagnosis (Mehes et al., 2001). Several molecular changes occur during this transformation of the normal tumor to malignant disseminated tumors. Other molecular changes enable disseminated tumors to relapse after chemotherapy. Most of the studies have focused on the genetic and molecular changes that happen in primary tumors, and a few have highlighted the changes that occur between the primary and the disseminated tumor in the coding region of the genome. However, no studies have reported the change in the lncRNA profile during the transformation of a benign tumor into disseminated tumors and during the relapse of those tumors after chemotherapy. We have explored publicly available next-generation sequencing datasets to identify probable lncRNA biomarkers associated with neuroblastoma. We observed differentially expressed lncRNAs in primary tumor, in DTCs, and in relapsed DTCs and then conducted epigenetic analysis of the identified lncRNAs and cross-validation in a cancer database. Our study thus provides a selective list of lncRNAs associated with neuroblastoma; these lncRNAs could be potent biomarkers for the identification of the onset or progression of high-risk or malignant neuroblastoma.

## Results

### Identification of Candidate lncRNA Markers in Disseminated Tumor Cells (DTCs) in the Bone Marrow and in Stage 4 Primary Tumor

**FIGURE 1:**
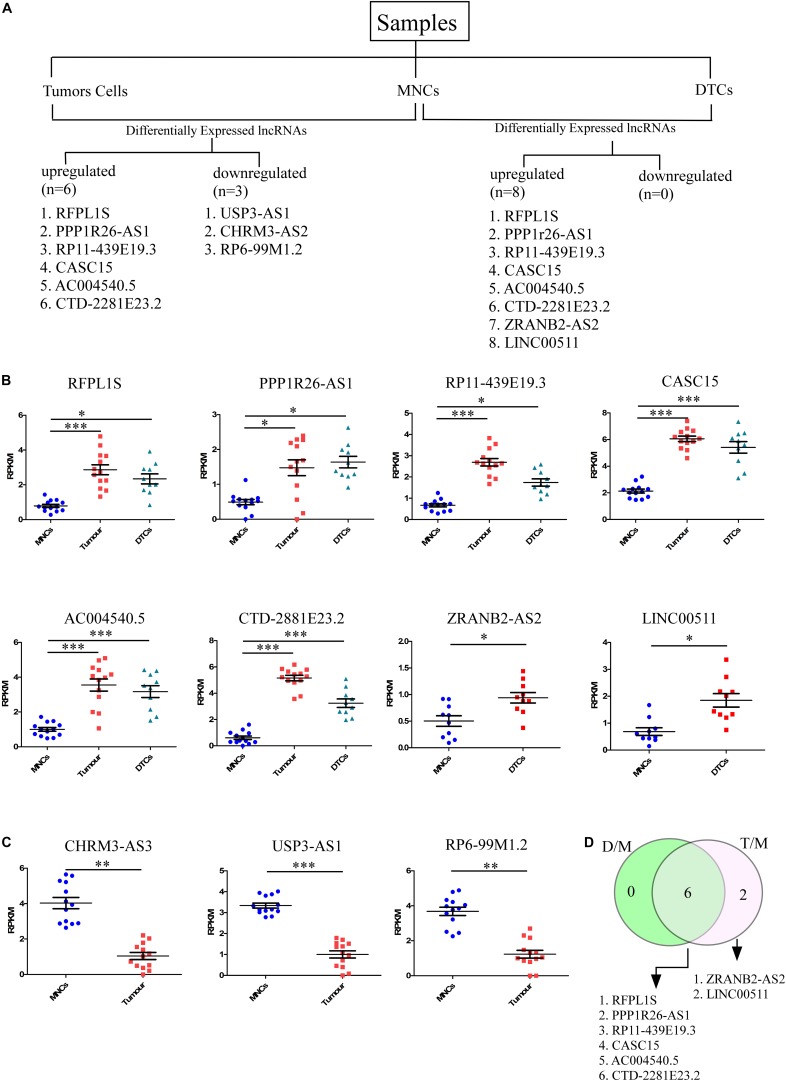
Identification of candidate lncRNA markers in disseminated tumor cells (DTCs) in bone marrow and in stage 4 primary tumor. **(A)** Workflow for the identification of lncRNA in DTCs and primary tumor. On the left, six upregulated and three downregulated lncRNAs were identified in the DTC sample when compared to matched MNCs from bone marrow. On the right, eight lncRNAs were upregulated and no lncRNAs were significantly downregulated in primary tumor samples when compared to matched MNCs. **(B)** Significantly upregulated lncRNAs *RFPL1S*, *PPP1R26-AS1*, *RP11-439E19.3*, *CASC15, AC004540.5*, and *CTD-2881E23.2* are commonly upregulated in both DTCs and primary tumor samples, while *ZRANB2-AS2* and *LINC00511* were two upregulated lncRNAs that are specific to DTCs. **(C)** *CHRM3-AS3*, *USP3-AS1*, and *RP6-99M1.2* were significantly downregulated in tumor samples. **(D)** Venn diagram showing common and tumor-specific lncRNAs in the overall differential expression analysis. D/M represents DELs between DTCs and MNCs, while T/M represents DELs between primary tumor and MNCs. ^∗^*p* ≤ 0.05, ^∗∗^*p* ≤ 0.005, ^∗∗∗^*p* ≤ 0.0005, two-tailed unpaired *t*-test with Welch’s correction.

To identify lncRNAs that could play an important role in neuroblastoma initiation and progression, an available RNA-seq dataset on neuroblastoma (GSE94035) (Rifatbegovic et al., 2018) was analyzed. Using the above repository of data, we identified candidate marker genes in stage M neuroblastoma patients. The differential expression of genes among transcriptomically distinct cell types was also available in this dataset, including (1) DTCs, GD2^*POS*^ tumor cells in the bone marrow (BM), which were isolated using magnetic bead-based enrichment, (2) MNCs, the corresponding non-tumor mononuclear cells isolated from bone marrow using the same magnetic bead-based enrichment method, and (3) Stage 4 primary tumor. The authors have identified many differentially expressed genes, but their major focus was on the top differentially coding genes, and they have not analyzed the data within the context of lncRNAs. The already available online dataset was therefore reanalyzed in this context to identify potential lncRNAs that could be important for neuroblastoma initiation and progression. The raw RNA-seq data in FASTQ formats was retrieved from the European Nucleotide Archive (ENA)^1^ with the respective accession number. We also analyzed the dataset from the NCBI GEO^2^ using the GSE94035 accession number. FASTQ files were not available for all of the samples, so we crosschecked the samples and selected 10 DTC samples, 13 tumor samples, and matched controls for the analysis (Figure 1A). Differential analysis revealed nine significantly differentially expressed (more than 2-fold change with corrected *p*-value) lncRNAs between tumor cells and matched MNCs. Of these, *RFPL1S* (*p* = 0.0001), *PPP1R26-AS1* (*p* = 0.03), *RP11-439E19.3* (*p* = 1.24 × 10^–6^), *CASC15* (*p* = 1.6 × 10^–7^), *AC004540.5* (*p* = 0.0002), and *CTD-2881E23.2* (*p* = 1.6 × 10^–9^) were found to be significantly upregulated (Figure 1B), while *USP3-AS1* (*p* = 1.78 × 10^–6^), *CHRM3-AS2* (*p* = 0.003) and *RP6-99M1.2* (*p* = 0.002) were significantly downregulated (Figure 1C).

Further, the differential expression between DTCs and matched MNCs was also studied, and eight differentially expressed lncRNAs were found, all of which were upregulated. Of these lncRNAs, six were common to tumor cells, *RFPL1S* (*p* = 0.019), *PPP1R26-AS1* (*p* = 0.008), *RP11-439E19.3* (*p* = 0.01), *CASC15* (*p* = 5.27 × 10^–5^), *AC004540.5* (*p* = 0.00042), and *CTD-2881E23.2* (*p* = 8.4 × 10^–6^), while two were unique to DTCs, *ZRANB2-AS2* (*p* = 0.033) and *LINC00511* (*p* = 0.03) (Figures 1B,D). Out of these lncRNAs, *CASC15* and *CTD-2881E23.2* were significantly upregulated in both DTCs and tumor and hence could be more important in the progression of neuroblastoma. Genome-wide association studies (GWAS) revealed several loci that were strongly associated with neuroblastoma development and aggressiveness. Interestingly, 6p22.3 is one of the many loci that was associated with neuroblastoma progression. *CASC15* lncRNA resides within this locus, and an SNP, rs6939340, which is present in the intron region of the *CASC15* lncRNA gene, is associated with the increased risk of neuroblastoma, particularly high-risk neuroblastoma (Pandey and Kanduri, 2015).

To understand the lncRNA transcriptomic differences between DTCs and tumor cells, we identified the significantly differentially expressed lnRNAs between them. We found only one upregulated lncRNA, *MAMDC2-AS1* (*p* = 0.02), and one downregulated lncRNA, *CHRM3-AS2* (*p* = 0.044) (data not shown). The low number of differentially expressed genes with significance but with a low *p*-value suggests that these two neuroblastoma cells are transcriptomically similar with respect to lncRNA expression.

### Three lncRNAs Are Specifically Differentially Expressed in Relapsed DTC Samples

**FIGURE 2:**
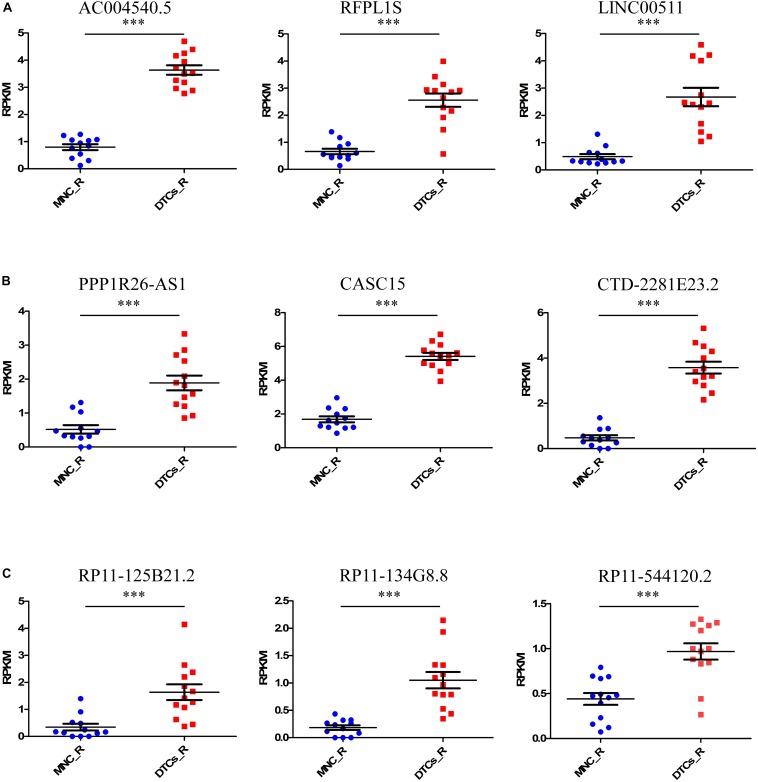
Identification of candidate lncRNA markers in relapsed DTCs. For this, we took relapsed samples indicated in GSE94035 to identify lncRNAs that are associated with tumor relapsing in neuroblastoma patients. Here we compared DELs in MNCs and DTCs of relapsed patients. **(A–C)** We found nine upregulated lncRNAs, *AC004540.5, CASC15*, *CTD-2281E23.2*, *RFPL1S*, *RP11-134G8.8*, *LINC00511*, *PPP1R26-AS1*, *RP11-125B21.2*, and *RP11-544I20.2*. Of these, the lncRNAs *RP11-134G8.8, RP11-125B21.2*, and *RP11-544I20.2* were specifically differentially expressed only in relapsed DTCs. ^∗∗∗^*p* ≤ 0.0005, two-tailed unpaired *t*-test with Welch’s correction.

Tumor relapse is a major problem in the treatment of neuroblastoma, and it is a major cause of death in stage 4 neuroblastoma. The identification of marker lncRNAs in relapsed tumor samples is important to understand the onset of the relapsed state of neuroblastoma. For this, we again studied the samples from the GSE94035 dataset, comparing relapsed DTC samples with matched MNCs. The differential expression of 13 available relapsed DTC samples and 12 control MNCs was studied to identify important lncRNAs that are responsible for the relapsed state of the tumor. We found nine upregulated lncRNAs, *AC004540.5* (*p* = 5.9 × 10^–8^), *CASC15* (*p* = 3.1 × 10^–8^), *CTD-2281E23.2* (*p* = 4.39 × 10^–5^), *RFPL1S* (*p* = 0.00044), *RP11-134G8.8* (*p* = 0.00079), *LINC00511* (*p* = 0.000824), *PPP1R26-AS1* (*p* = 0.0061), *RP11-125B21.2* (*p* = 0.025), and *RP11-544I20.2* (*p* = 0.039) and no downregulated lncRNAs (Figures 2A–C). Of the former, *RP11-134G8.8, RP11-125B21.2*, and *RP11-544I20.2* were the lncRNAs that were specifically differentially expressed in relapsed DTCs. Interestingly, other six lncRNAs were differentially expressed in all types of tumors (stage 4 primary tumor, DTCs, and relapsed DTCs), indicating their importance in the onset of the tumor and that they could be important biomarkers for the identification of neuroblastoma oncogenesis.

### Epigenetic Analysis of Differentially Expressed lncRNAs in a Cross-Sectional Dataset of the Neuroblastoma Cell Line

Epigenetic changes play a crucial role in the progression of neuroblastoma. Epigenetic changes or mutations of epigenetic regulators affect the global transcriptional regulatory circuitry, leading to neuroblastoma oncogenesis (Durinck and Speleman, 2018). To understand whether these differentially expressed lncRNAs experience epigenetic changes, we explored a dataset that dealt with the epigenetic changes in cases of high-risk neuroblastoma drawn from a report that presents the dynamic chromatin and transcriptional landscape of *MYCN* perturbation in neuroblastoma (Zeid et al., 2018). The authors performed ChIP-seq analysis for *MYCN* and epigenetic regulators such as H3K4me3 (for active promoters), H3K27me3 (for repressive promoters), ATAC-seq, and other transcription regulators such as BRD4 and Pol II. They also performed ChIP-seq analysis in various cell lines, including the BE(2)-C neuroblastoma cell line.

**FIGURE 3:**
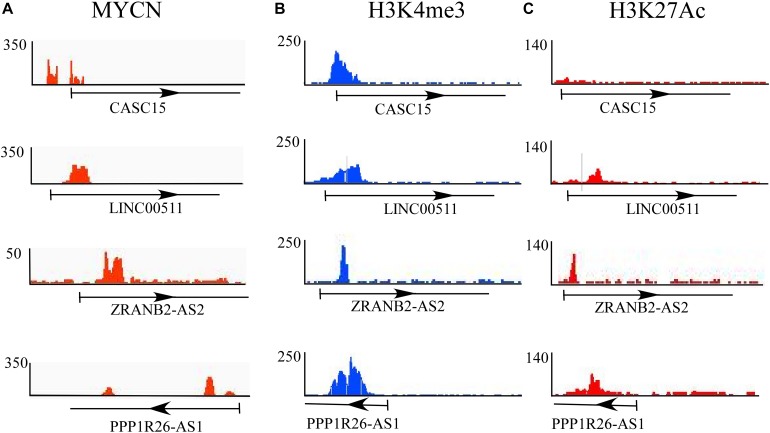
Epigenetic analysis of differentially expressed lncRNAs. ChIP-seq analysis of **(A)** MYCN and epigenetic regulators such as **(B)** H3K4me3 (for active promoters) and **(C)** H3K27me3 (for repressive promoters). Of 11 identified lncRNAs, only four, *CASC15*, *LINC00511*, *ZRANB2-AS2*, and *PPP1R26-AS1*, show *MYCN* and epigenetic marker bindings.

In this study, the epigenetic changes (H3K4me3 and H3K27me3) and *MYCN* bindings in all 11 significantly differentially expressed lncRNAs in BE(2)-C were checked, since these cell lines were derived from bone marrow and are comparable to DTCs. Of these 11 identified lncRNAs, only four lncRNAs, *CASC15*, *LINC00511*, *ZRANB2-AS2*, and *PPP1R26-AS1*, showed *MYCN* and epigenetic marker bindings (Figures 3A–C). Among those four, *CASC15* showed only *MYCN* and active histone mark H3K4me3 bindings, while the others showed binding of both active and repressive markers. *CASC15* clearly exhibited only active histone marks but not the repressed state, which represents its transcriptional state in neuroblastoma. The presence of both the markers represents the “poised” transcriptional state, which is a property of developmental genes. Neuroblastoma is a disease that is believed to emanate due to altered differentiation and development of the neural crest cells (Brodeur, 2003; Kamijo and Nakagawara, 2012), and the poised state of these genes indicates the developmental characteristics of the tumor.

### Cross-Validation of Differentially Expressed lncRNA Expression and Survival Probability in a Cancer Database

**FIGURE 4:**
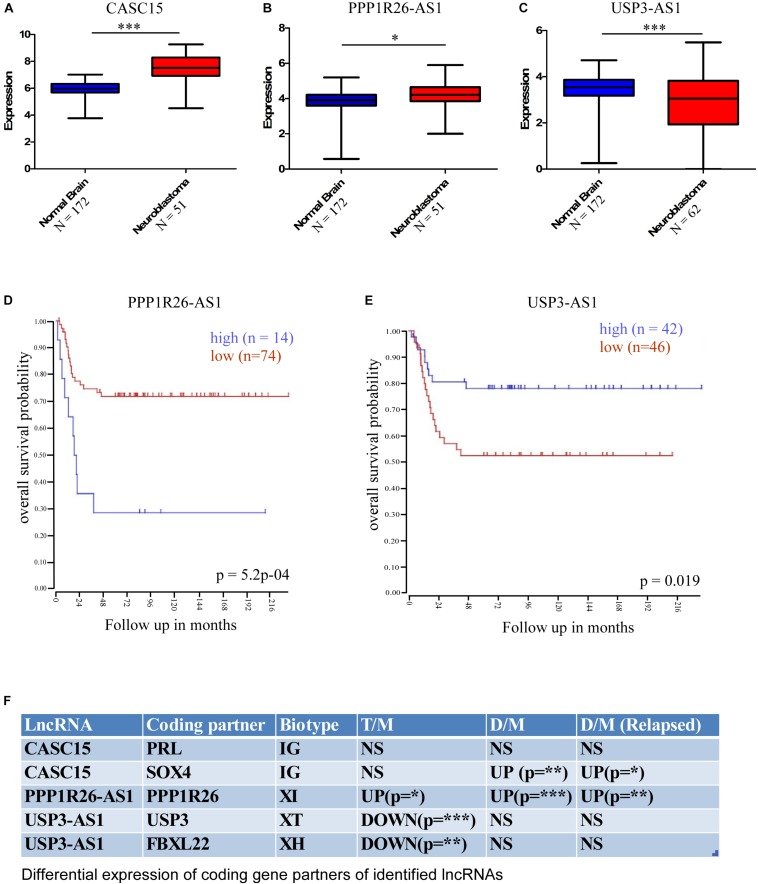
Cross-validation of differentially expressed lncRNA expression in the cancer database and differential expression of lncRNA-associated protein-coding partners. This database revealed that the expressions of three lncRNAs were significantly altered in neuroblastoma samples; two were upregulated, **(A)** *CASC15* (Normal brain, *N* = 172 and neuroblastoma samples [Hiyama], *N* = 51), **(B)** *PPP1R26-AS1* (Normal brain, *N* = 172 and neuroblastoma samples [Hiyama], *N* = 51) and one was downregulated, **(C)** *USP3-AS* (Normal brain, *N* = 172 and neuroblastoma samples [Delattre], *N* = 64) (^∗^*p* ≤ 0.05, ^∗∗∗^*p* ≤ 0.0005, unpaired two-tailed *t*-test with Welch’s correction). Kaplan–Meier survival plots of **(D)** upregulated gene *PPP1R26-AS1* (*p* = 5.2^∗^10^– 4^, number of samples with high expression = 14, number of samples with low expression = 74) and **(E)** downregulated gene *USP3-AS1* (*p* = 0.019, number of samples with high expression = 42, number of samples with low expression = 46). Data were obtained from http://hgserver1.amc.nl/cgi-bin/r2/main.cgi. **(F)** Table showing differential expression of coding-gene partners of identified lncRNAs. LncRNA *CASC15* has two coding partners, *PRL* and *SOX4*, and *PRL* was not significantly altered, while *SOX4* was significantly upregulated in DTCs and relapsed DTCs. *PPPIR26-AS1*-associated coding gene *PPP1R26* was significantly upregulated in all three types of neuroblastoma samples. *USP3-AS1*-associated coding genes *USP3* and *FBXL22* were significantly downregulated only in primary tumor samples (^∗^*p* ≤ 0.05, ^∗∗^*p* ≤ 0.005, ^∗∗∗^*p* ≤ 0.0005 unpaired two-tailed *t*-test with Welch’s correction). D/M represents DELs between DTCs and MNCs, T/M represents DELs between primary tumor and MNCs, and D/M (relapsed) represents DELs between relapsed DTCs and relapsed MNCs. IG, Intergenic lncRNA, XH, Divergent or Antisense Head-to-Head lncRNAs, XT, Convergent or Antisense Tail-to-Tail lncRNAs, XI, Antisense Inside lncRNAs.

Further, for cross-validation of these differentially expressed lncRNAs, we checked their expression in a publicly available database. We used the R2 Expression Analysis and Visualization Platform^3^ to analyze their expression in normal brain tissue and neuroblastoma tissue samples [named as per the R2 database: Normal brain regions – Berchtold (*n* = 172), Hiyama (*n* = 51), and Tumor Neuroblastic mixed – Delattre (*n* = 64)]. This database revealed that the expression of three lncRNAs was altered significantly in neuroblastoma samples; two were upregulated, namely *CASC15* (*p* < 0.0001, unpaired two-tailed *t*-test with Welch’s correction) and *PPP1R26-AS1* (*p* < 0.05, unpaired two-tailed *t*-test with Welch’s correction), and one was downregulated, *USP3-AS1* (*p* < 0.0001, unpaired two-tailed *t*-test with Welch’s correction) (Figures 4A–C). The overall analysis revealed that the *CASC15* and *PPP1R26-AS1* lncRNAs are significantly upregulated in all tumor cells (primary stage 4 tumor, DTCs, and relapsed DTCs) and also in the publicly available database, which makes them strong candidates for marker lncRNA genes for the identification of neuroblastoma progression.

To understand the effect of altered expression on patient survival, we used the survival probability dataset of the R2 Genomics and Visualization Platform^4^. The survival probability was estimated with respect to the altered expression of these identified lncRNAs using the “Kaplan Meier by gene expression” analysis function. This analysis revealed that the expression of only two lncRNAs is significantly associated with overall survival probability. Of these, one was upregulated, *PPP1R26-AS1* (*p* = 5.2 × 10^–4^, number of samples with high expression = 14, number of samples with low expression = 74), and the other was downregulated, USP3-AS1 (*p* = 0.019, number of samples with high expression = 42, number of samples with low expression = 46) (Figures 4D,E).

It is now well established that lncRNAs can interact with the DNA of nearby protein-coding genes and affect their transcription (Luo et al., 2016; Prajapati et al., 2019). Therefore, the functional role of lncRNAs can be speculated upon by studying their coding gene partners. To understand the functional role of the identified lncRNAs in neuroblastoma, we checked the differential expression of the lncRNA-associated coding genes. According to a recent classification, lncRNA biotypes can be broadly classified into two groups: Genic, lncRNA (<5 kb to a coding gene), and intergenic, IG (>5 kb to a coding gene). Genic lncRNAs are further categorized into six biotypes: Divergent or Antisense Head-to-Head (XH), Convergent or Antisense Tail-to-Tail (XT), Antisense Outside (XO), Antisense Inside (XI), Sense Downstream (SD), and Sense Upstream (SU) (Luo et al., 2016). We have identified five lncRNA-associated coding partners that are near (around the 5 kb region) to cross-validated lncRNAs *CASC15, PPP1R26-AS1*, and *USP3-AS1*, which belong to different classes/biotypes of lncRNAs. The *PRL* and *SOX4* protein-coding genes are associated with *CASC15*, *PPP1R26* is associated with *PPP1R26-AS1*, and *USP3* and *FBXL22* are associated with *USP3-AS1*. Interestingly, the *PPP1R26-AS1-*associated gene *PPP1R26* was differentially expressed in all of the conditions [Tumor vs. MNCs, DTCs vs. MNCs, and DTCs (relapsed) vs. MNCs (relapsed)], whereas *USP3-AS1*-associated coding genes *USP3* and *FBXL22* were differentially expressed only in primary neuroblastoma tumor. Further, only one of the two coding genes associated with *CASC15* lncRNA, *SOX4*, was differentially expressed in DTCs and the relapsed form of DTCs (Figure 4F).

## Discussion

In recent studies, the role of lncRNAs in cancer research has become more important, mainly in prostate and breast cancers. Further speculation on the possible role of lncRNAs in neuroblastoma should also be explored. Genetic predisposition in neuroblastoma has been well studied, and many loci associated with neuroblastoma have been identified. These include deletion of some (1p, 3p, and 11q) and gain of others (1q, 2p, and 17q), and these covered almost 50% of neuroblastoma cases (Caron, 1995; Maris et al., 1995; Castel et al., 2007; Schleiermacher et al., 2012; Theissen et al., 2014). Predisposing variant/mutations on non-coding genomic regions commonly go unnoticed, but some variants present on the genomic loci contain lncRNA genes that have been associated with high-risk neuroblastoma. These lncRNAs include *CASC15* (alias *LINC00340*) and *NBAT1* (alias *CASC14*), and their role in neuroblastoma carcinogenesis has been further elucidated (Diskin et al., 2012; Molenaar et al., 2012; Pandey et al., 2014). Similar to other transcriptomic analyses in cancer, the presence of the *T-UC.3004* (Watters et al., 2013), *linc00467* (Atmadibrata et al., 2014), *NBAT1* (Mondal et al., 2018), and *Pauppar* (Vance et al., 2014) lncRNAs has been reported during the pathogenesis of neuroblastoma.

A highly mutational landscape is not predominantly associated with neuroblastoma, which is also reasonably true for other pediatric cancers (Durinck and Speleman, 2018). Therefore, the role of epigenetic changes in relation to neuroblastoma cannot be ignored. Recently, a report emphasized the importance of epigenetic changes such as altered chromatin regulator activity, chromatin remodeling, and DNA methylation in tumorigenesis (Durinck and Speleman, 2018). Beside many epigenetic changes, histone methylation, especially H3K4me3 and H3K27me3 methylation, played an important role in the altered promoter activity in tumorigenesis. For example, WDR5 is a protein that exists as part of many chromatin regulatory complexes, and it is a histone H3K4 presenter that has been reported in association with neuroblastoma (Sun et al., 2015). WDR5 is a principal component of the H3K4me3 writer complex MLL–SET1 and interacts with MYC oncoprotein, allowing MYC to select its target genes and alter their expression epigenetically in cancer (Thomas et al., 2015). In neuroblastoma, high levels of WDR5 expression are associated with poor survival in primary neuroblastoma cases (Sun et al., 2015). Unlike H3K4me3, which is associated with gene activation, methylation of H3K27 leads to gene suppression and has been linked to neuroblastoma tumor (Henrich et al., 2016). The methylation of H3K27 is catalyzed by PRC2 and is reported to act either as a tumor suppressor or oncogene. In the case of neuroblastoma, tumors with high amplification of MYCN show hyperactivation of PRC2-associated components such as EZH2, EED, and RBBP7 with respect to non-MYCN-amplified neuroblastoma cases (Henrich et al., 2016). In another study, the overexpression of EZH2 in neuroblastoma was found to be associated with poor patient prognosis, and PRC2 mediated the silencing of tumor suppressor genes like *CASZ1* through epigenetic changes (Wang et al., 2012). In this study, we aimed to understand the epigenetic status of identified DELs in neuroblastoma. Despite the differential expression of these lncRNAs, epigenetic analysis is relevant and important for the identification of potential biomarkers with respect to neuroblastoma. We chose to conduct epigenetic analysis of the BE(2)-C neuroblastoma cell line because this cell line is similar to bone marrow-derived DTCs. From all of the DELs, we filtered those lncRNAs that have MYCN binding, as this makes them strong candidate neuroblastoma biomarkers. Further binding of other epigenetic markers, H3K4me3 and H3K27me3, further strengthens the candidature of these identified lncRNAs as biomarkers for neuroblastoma.

**FIGURE 5:**
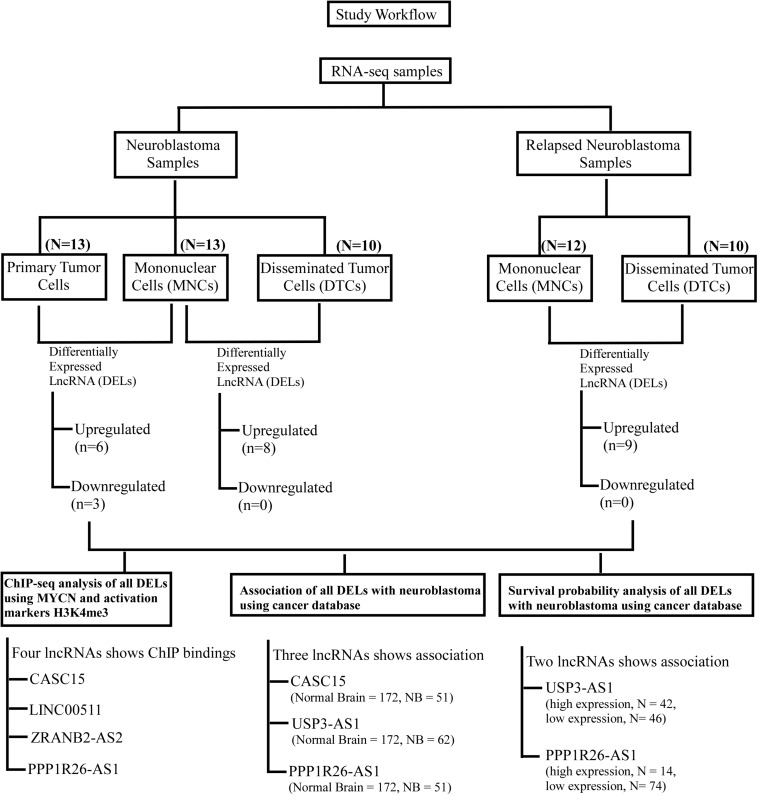
Workflow of the study to identify lncRNAs as biomarkers for neuroblastoma. This workflow is segregated in two parts: the upper part shows the identification of differentially expressed lncRNAs in neuroblastoma and the relapsed form of neuroblastoma, and the lower panel shows the cross-validation of these DELs using ChIP-seq analyses (MYCN, H3K4me3, and K3K27me3) and their association with neuroblastoma using cancer databases. Finally, survival probability analysis of neuroblastoma patients with respect to these DEL expressions.

In our cross-sectional study, an attempt has been made to identify lncRNAs that could be strongly associated with neuroblastoma and are important for understanding the pathogenesis of neuroblastoma. We also tried to check the epigenetic changes of these lncRNAs genes and cross-validate them with a cancer database. After cross-sectional analysis, three lncRNAs, *CASC15*, *PPP1R26-AS1* and *USP3-AS1*, were found to be significantly altered in different datasets compared to other lncRNAs (Figure 5). These results indicate that the above lncRNAs could be good RNA biomarkers and may play an important role in the diagnosis of neuroblastoma. Recently Genome-Wide Association Studies (GWAS) have revealed many genomic loci associated with neuroblastoma. One of the loci, 6p22.3, harbors the *CASC15* gene, and variants within this gene is associated with the progression of neuroblastoma (Maris et al., 2008; Russell et al., 2015). The *CASC15* lncRNA is transcribed in an antisense manner with another non-coding gene *NBAT1*, which is also associated with neuroblastoma progression (Pandey et al., 2014; Mondal et al., 2018). In one study, lower expression of one of the variants of *CASC15* was correlated with poor prognosis in neuroblastoma patients (Mondal et al., 2018), while, in contrast, we observed a higher expression in neuroblastoma samples. Overexpression of *CASC15* is also associated with the progression and phenotype switching of melanoma (Russell et al., 2015). However, the understanding of the functional role of *CASC15* and its plausible role in neuroblastoma is still in its preliminary stage. Still, Gene Ontology study of *CASC15* shows its involvement in the regulation of genes that contribute to neural crest development (Russell et al., 2015). This points to the need for functional characterization of these lncRNAs. Another identified lncRNA, *PPP1R26-AS1*, is reported in association with breast cancer in GWAS using the Gene Expression Omnibus (GEO) and Cancer Genome Atlas (TCGA) databases (Xu et al., 2017). Moreover, *PPP1R26-AS1* and some other lncRNAs have been shown to be associated with luminal B subtypes of breast cancer (Xu et al., 2017; Li et al., 2018). Interestingly, the protein-coding partner of *PPP1R26-AS1* lncRNA, *PPP1R26*, was also differentially expressed in all sets of neuroblastoma, which could shed light on the molecular function of the identified lncRNA. Exogenous expression of *PPP1R26* (*KIA0649*) in NIH3T3 fibroblasts caused tumor formation and enhanced colony formation as well as allowing anchorage-independent growth (Yang et al., 2005). However, the association or any functional analysis of the third of the identified lncRNAs, *USP3-AS1*, has not been reported with any cancer. In this context, the identification of a few lncRNAs in cross-sectional studies of neuroblastoma prognosis in the present study is of significance. These lncRNAs could possibly play an important role in the identification of neuroblastoma. In the future, functional analyses of these lncRNAs will help in understanding the role of lncRNA in the cellular and molecular pathogenesis of neuroblastoma progression.

## Methods

### Public Database Portals and Dataset Analyses

#### Accession Numbers and Number of Samples Used

The RNA-seq and ChIP-seq data used in this paper had previously been deposited in the NCBI Gene Expression Omnibus (GEO) database and R2 GeneSet Clustering Analysis portal (hgserver1.amc.nl/cgi-bin/r2/main.cgi). Data for primary tumors, DTCs, and MNCs were obtained from GEO number GSE94035 (Rifatbegovic et al., 2018). FASTQ files of this data can be obtained from the European Nucleotide Archive (ENA see text footnote 1) with BioProject number “PRJNA368627”. From these repositories, we were able to gather data on 62 samples of which 13 were primary tumor samples (Patient IDs: P5, P6 P44, P47, P48, P50, P52, P53, P54, P55, P56, P57, and P62), 13 were MNCs (Patient IDs: P1, P2, P3, P4, P6, P10, P27, P28, P30, P31, P33, P59, and P60), 10 were DTCs (Patient IDs: P1, P4, P10, P27, P33, P36, P37, P39, P42, and P61), and 12 samples of MNCs and 13 DTCs from relapsed samples, respectively (Patient IDs of relapsed MNCs: P7, P11, P12, P13, P15, P20, P21, P22, P23, P24, P33, and P34; Patient IDs of relapsed DTCs: P7, P11, P14, P15, P16, P17, P18, P20, P22, P23, P24, P25, and P26).

Analysis of ChIP-seq was done using the data of neuroblastoma cell line BE(2)-C from GEO number GSE80154. Accession numbers for different antibodies are listed in Table 1.

**TABLE 1:** Accession numbers for antibodies used in ChIP-seq analysis.

**Cell Types**	**Antibody**	**Accession number**
BE2C	MYCN	GSE80154 (GSM2113521)
BE2C	H3K27Ac	GSE80154 (GSM2113518)
BE2C	H3K4me3	GSE80154 (GSM2113519)
BE2C	Input	GSE80154 (GSM2113520)

### RNA-seq Analysis and Extraction of lncRNA

We retrieved raw RNA-seq data in FASTQ formats from the European Nucleotide Archive (ENA see text footnote 1) with the GSE94035 accession numbers. The quality of the FASTQ files was estimated by using the *“FastQC”* tools in the Galaxy web portal^5^, and low-quality readings were trimmed down using Galaxy’s *“filter by quality”* tool. For high-quality reads, the Phred quality cut-off value was set at 30, and the percentage of bases in the sequence that had a quality of ≥30 were taken as 90. Transcript-level expression analysis of the RNA-seq experiment was performed with HISAT, StringTie, and the R-based visualization tool, Ballgown (Pertea et al., 2016). A human genome (hg19) indexed file was generated using the *hisat2-build* script in HISAT2 (Kim et al., 2015). The quality-checked FASTQ files were aligned using HISAT2 with the default parameters of the *hisat2* script. The output SAM files were then converted and sorted into BAM files using SAMtools. StringTie was used to assemble and quantify the expressed genes and transcripts. Differential expression analysis was performed using R-based packages that included Ballgown for FPKM abundance estimation and statistical analysis of significant differences between the groups. *RSkittleBrewer* was used for setting up color, *genefilter* for fast calculation of means and variance, *dplyr* for sorting and arranging results, and *devtools* for reproducibility and installing packages. To obtain cRNA information from whole differentially expressed files, we first downloaded the transcript IDs from HUGO for lncRNAs and joined these two data files with the lncRNA IDs as the common attribute in both of the files using the Galaxy tool *“join two datasets”.* The output file contained only the RPKM values of lncRNAs. The fold changes of gene expression between the groups were again checked by two-tailed test and Bonferroni p-correction.

### ChIP-seq Analysis

Data from GSE80154 were used as provided by the GEO portal without additional processing or normalization. We used individual files in the WIG format for MYCN and epigenetic factors H3K4me3 and H3K27Ac. The accession number of these files are mentioned in Table 1. For visualization of ChIP-seq peak files, we used the Integrative Genomic Viewer (IGV), and the peak images were saved for the gene of interest.

### Statistical Analysis

The data are represented as mean ± SD. The significance values of the comparisons between the primary tumor, DTC/relapsed DTC, and MNC/relapsed MNC groups were computed using paired two-tailed Student’s *t*-test with Bonferroni’s correction. *P* ≤ 0.05 was considered as the minimum for statistically significance. For the cancer database, we used an unpaired two-tailed student’s *t*-test with Welch’s correction.

## Data Availability Statement

Publicly available datasets were analyzed in this study. This data can be found here: GSE94035 and GSE80154.

## Author Contributions

PS and SS conceived and directed this study. BP, MeF, and MF performed the bioinformatics work. MF and MK helped in the critical analysis of the results. PS, BP, and MeF wrote the manuscript with the collaboration of the other authors. MF and MK performed the critical editing of the manuscript. All of the authors discussed the results and commented on the manuscript.

## Conflict of Interest

The authors declare that the research was conducted in the absence of any commercial or financial relationships that could be construed as a potential conflict of interest.

## Footnotes

1 https://www.ebi.ac.uk/ena

2 https://www.ncbi.nlm.nih.gov/geo/

3 http://hgserver1.amc.nl/cgi-bin/r2/main.cgi

4 http://r2.amc.nl

5 https://usegalaxy.org/
